# Multiscale spatial genetic structure within and between populations of wild cherry trees in nuclear genotypes and chloroplast haplotypes

**DOI:** 10.1002/ece3.5628

**Published:** 2019-09-04

**Authors:** Teruyoshi Nagamitsu, Kato Shuri, Satoshi Kikuchi, Shinsuke Koike, Shoji Naoe, Takashi Masaki

**Affiliations:** ^1^ Hokkaido Research Center Forestry and Forest Products Research Institute Forest Research and Management Organization Sapporo Japan; ^2^ Tama Forest Science Garden Forestry and Forest Products Research Institute Forest Research and Management Organization Hachioji Japan; ^3^ Forestry and Forest Products Research Institute Forest Research and Management Organization Tsukuba Japan; ^4^ Institute of Agriculture Tokyo University of Agriculture and Technology Fuchu Japan; ^5^ Tohoku Research Center Forestry and Forest Products Research Institute Forest Research and Management Organization Morioka Japan

**Keywords:** *Cerasus jamasakura*, *Cerasus leveilleana*, kinship coefficient, microsatellite, *Padus grayana*, seed dispersal, tree distribution

## Abstract

Spatial genetic structure (SGS) of plants mainly depends on the effective population size and gene dispersal. Maternally inherited loci are expected to have higher genetic differentiation between populations and more intensive SGS within populations than biparentally inherited loci because of smaller effective population sizes and fewer opportunities of gene dispersal in the maternally inherited loci. We investigated biparentally inherited nuclear genotypes and maternally inherited chloroplast haplotypes of microsatellites in 17 tree populations of three wild cherry species under different conditions of tree distribution and seed dispersal. As expected, interpopulation genetic differentiation was 6–9 times higher in chloroplast haplotypes than in nuclear genotypes. This difference indicated that pollen flow 4–7 times exceeded seed flow between populations. However, no difference between nuclear and chloroplast loci was detected in within‐population SGS intensity due to their substantial variation among the populations. The SGS intensity tended to increase as trees became more aggregated, suggesting that tree aggregation biased pollen and seed dispersal distances toward shorter. The loss of effective seed dispersers, Asian black bears, did not affect the SGS intensity probably because of mitigation of the bear loss by other vertebrate dispersers and too few tree generations after the bear loss to alter SGS. The findings suggest that SGS is more variable in smaller spatial scales due to various ecological factors in local populations.

## Graphical abstract

Spatial genetic structure (SGS) of plants may differ between biparentally and maternally inherited loci and vary among spatial scales. We found more intensive SGS in maternally inherited loci in a larger spatial scale although SGS in both loci is more variable in a smaller spatial scale due to various local factors, such as tree aggregation.

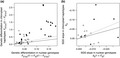

## 1 INTRODUCTION

Gene flow mediated by pollen and seed dispersal affects the genetic structure in plant populations. Pollen dispersal transfers only a male gamete, while seed dispersal transfers an embryo derived from both male and female gametes. Thus, seed dispersal is twice as effective as pollen dispersal in gene flow although pollen is often dispersed over longer distance than seeds (Epperson, 2007). Highly polymorphic genetic markers, such as microsatellites, in both nuclear and organelle genomes are useful to estimate gene flow. To distinguish seed dispersal from pollen dispersal, maternal tissues of dispersed seeds and maternally inherited genetic markers are available for genetic analyses of gene flow (Broquet & Petit, 2009; Hamrick & Trapnell, 2011). Assignment of mother plants with identical genotypes of maternal tissues of dispersed seeds directly estimates seed dispersal (Browne, Ottewell, Sork, & Karubian, 2018; Grivet, Robledo‐Arnuncio, Smouse, & Sork, 2009). On the other hand, genetic markers with uniparental and biparental inheritance indirectly estimate the contribution of pollen and seed dispersal to gene flow (Chybicki, Dering, Iszkuło, Meyza, & Suszka, 2016; Ennos, 1994; Hamilton & Miller, 2002; Petit, Duminil, & Fineschi, 2005).

Genetic differentiation between populations is expected to differ between genetic markers under different modes of inheritance (Ennos, 1994; Petit et al., 2005). Maternally inherited loci are predicted to show higher interpopulation genetic differentiation than biparentally inherited loci due to smaller effective population sizes and fewer opportunities of gene flow in the former loci (Hamilton & Miller, 2002; Appendix S1). The ratio of pollen and seed flow between populations can be estimated from the ratio of interpopulation genetic differentiation in maternally and biparentally inherited genetic markers (Ennos, 1994; Appendix S1). The median of pollen‐to‐seed‐flow ratio estimates was 17 in various coniferous and angiosperm taxa, indicating more extensive pollen flow than seed flow between plant populations (Petit et al., 2005).

In addition to genetic differentiation between populations, spatial genetic structure (SGS) within populations reflects gene dispersal in a local scale (Epperson, 2007). Fine‐scale SGS is often described as declining genetic relatedness with increasing spatial distance between individuals in a local area (Vekemans & Hardy, 2004) and quantified as linear regression of the genetic relatedness on the logarithm of spatial distance (Furstenau & Cartwright, 2016). The index *Sp* calculated from the regression slope and the relatedness between neighbors is often used to compare SGS intensity among populations (Vekemans & Hardy, 2004). The SGS intensity is expected to differ between genetic markers under different modes of inheritance (Chybicki et al., 2016). Maternally inherited loci are expected to show higher SGS intensity than biparentally inherited loci due to lower effective population densities and fewer opportunities of gene dispersal in the former loci (Hardy & Vekemans, 1999; Appendix S1). The ratio of pollen and seed dispersal area can be inferred from the ratio of regression slopes in maternally and biparentally inherited loci (Chybicki et al., 2016; Appendix S1). Because of the low polymorphism of maternally inherited loci within populations, fine‐scale SGS has rarely been compared between genetic markers with maternal and biparental inheritance. Variants of a cytoplasmic male sterility gene (de Cauwer, Dufay, Cuguen, & Arnaud, 2010) and chloroplast haplotypes (Latouche‐Hallé, Ramboer, Bandou, Caron, & Kremer, 2003; Silvestrini, McCauley, Zucchi, & Santos, 2015; Torroba‐Balmori et al., 2017), which are maternally inherited, exhibited more intensive SGS than biparentally inherited nuclear genotypes.

As theoretically expected, empirical studies of plant species suggest that both genetic differentiation between populations and SGS intensity within populations are associated with pollen and seed dispersal systems (Duminil et al., 2007; Hamrick & Godt, 1996; Hardy et al., 2006; Nybom, 2004; Vekemans & Hardy, 2004). Dioecious or outcrossing species, which seem to have wider ranges of pollen dispersal, tend to have lower genetic differentiation (Duminil et al., 2007; Hamrick & Godt, 1996; Nybom, 2004) and lower SGS intensity (Vekemans & Hardy, 2004) in nuclear markers. Species with seed dispersal by gravity, which seem to have narrower ranges of seed dispersal, tend to have higher genetic differentiation in organelle markers (Duminil et al., 2007) and higher SGS intensity in nuclear markers (Hardy et al., 2006). The SGS intensity in nuclear markers increases as local plant density increases because higher density may result from shorter seed dispersal and may result in shorter pollen dispersal under the same effective population size (Epperson, 2007; Hardy et al., 2006). In addition to pollen and seed dispersal systems, various factors, such as perenniality (Duminil et al., 2007), geographic distributional range (Duminil et al., 2007), colonization history (Hamrick & Trapnell, 2011), affect the genetic differentiation or SGS intensity. Among populations within species, habitat conditions, such as disturbance (Rico & Wagner, 2016) and fragmentation (Yamagishi, Tomimatsu, & Ohara, 2007), affect the SGS intensity in nuclear markers. Although these effects on interpopulation genetic differentiation or within‐population SGS intensity have often been evaluated using either nuclear or organelle markers, both markers have been rarely used together to evaluate those factors.

Wild cherry species of the genera *Cerasus* and *Padus* are common trees in mountainous regions on the Japanese mainland (Iwasaki, Aoki, Seo, & Murakami, 2012; Tsuda, Kimura, et al., 2009). Many microsatellites in both nuclear and chloroplast genomes are available in the cherry species (Cho, Yoon, & Kim, 2018; Kato et al., 2014). As in most angiosperms, chloroplast genomes are inherited maternally in *Cerasus* species (Brettin, Karle, Crowe, & Lezzoni, 2000). Thus, SGS in cherry tree populations can be compared between maternally and biparentally inherited loci using chloroplast and nuclear microsatellites, respectively. We expected higher interpopulation genetic differentiation and more intensive within‐population SGS in chloroplast haplotypes than in nuclear genotypes as mentioned above.

*Cerasus* and *Padus* species are predominantly outcrossing because of their self‐incompatibility (Ushijima et al., 2004). These species have similar pollinators, including various insects dominated by bees (Nagamitsu, Shuri, Taki, Kikuchi, & Masaki, 2016), and similar seed dispersers, including birds and mammals (Fujitsu, Masaki, Naoe, & Koike, 2016; Koike, Morimoto, Goto, Kozakai, & Yamazaki, 2008). Thus, both species are expected to have similar ratios of pollen and seed flow between populations. Also within populations, both species are expected to have similar ratios of pollen and seed dispersal area. *Cerasus* and *Padus* species have different life history traits, such as regeneration strategies. *Padus* species have more restricted regeneration habitats than *Cerasus* species (Masaki, 2002), which may result in different patterns of spatial tree distribution between the genera. Fine‐scale SGS in nuclear genotypes and chloroplast haplotypes may be associated with spatial tree distribution (Epperson, 2007; Hardy et al., 2006). Among seed dispersers of wild cherry species, Asian black bears, *Ursus thibetanus* G. Cuvier, are the most effective dispersers on the Japanese mainland (Koike, Kasai, Yamazaki, & Furubayashi, 2008; Koike et al., 2011; Koike, Morimoto, et al., 2008; Naoe et al., 2016). Populations of Asian black bears had declined until the 20th century due to human impacts (Oi & Yamazaki, 2006), and they have been locally extinct for about 150 years in some mountainous regions on the Japanese mainland (Hanai, 1980; Tsujino, Ishimaru, & Yumoto, 2010). Thus, the seed disperser loss may reduce seed dispersal relatively to pollen dispersal, resulting in a difference in fine‐scale SGS between nuclear genotypes and chloroplast haplotypes.

To study the expected higher interpopulation genetic differentiation and higher within‐population SGS intensity in chloroplast haplotypes than in nuclear genotypes, we investigated 17 tree populations of three wild cherry species, *Cerasus jamasakura* (Koidz.) H. Ohba, *C. leveilleana* (Koehne) H. Ohba, and *Padus grayana* (Maxim.) C. K. Schneider. Next, we evaluated whether the spatial distribution of trees and the loss of seed dispersers affect fine‐scale SGS in these populations.

## 2 METHODS

### 2.1 Study sites and sampling

**Figure 1:**
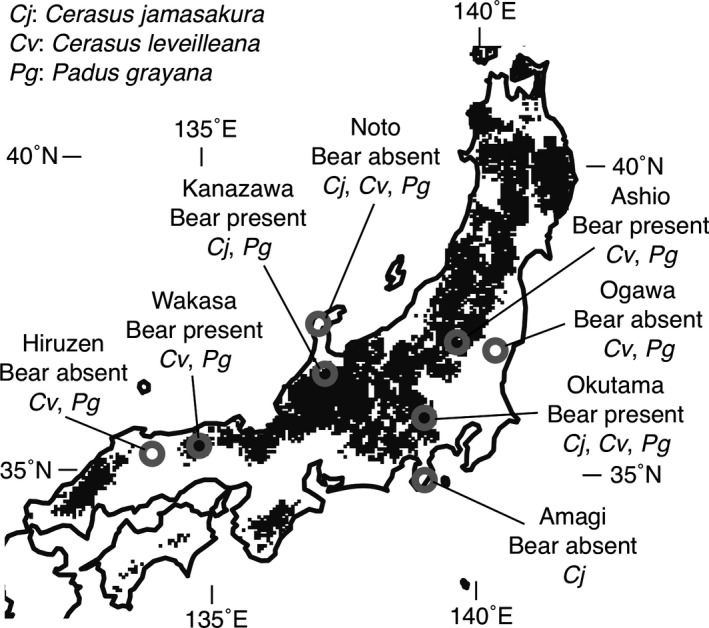
Study site locations and cherry species on the Japanese mainland. Gray circles indicate study sites. Black pixels indicate bear distribution in 1978

We selected four sites where bears were present and four sites where bears were absent in mountainous regions on the Japanese mainland (Figure 1) on the basis of the national survey of *U. thibetanus* distribution in 2003 (Ministry of Environment, 2004). At bear‐absent sites, Asian black bears also had not been recorded in 1978 (Ministry of Environment, 2004) and had been rare since at least the middle of the 19th century (Hanai, 1980; Tsujino et al., 2010). Thus, most of cherry trees at bear‐absent sites had been regenerated without seed dispersal by the bears.

Of the eight sites, we sampled *C. jamasakura* at four sites, *C. leveilleana* at six sites, and *P. grayana* at seven sites because some species were rare at some sites (Table 1). Thus, we investigated 17 populations of the three species at the eight sites. Linear sampling, which is associated with a linear transect, such as a river, road, or trail, was performed as well as random sampling to estimate SGS (Oyler‐McCance, Fedy, & Landguth, 2013). We sampled trees along one or two routes at each site (Figure S1) because random sampling from an entire area of seed dispersal ranges would require an impractical effort. For *C. jamasakura* and *C. leveilleana* populations at Okutama site and *C. leveilleana* population at Ogawa site, we selected two routes because their habitats were fragmented (Figure S1). The total length of routes at each site ranged from 1.3 to 12.8 km depending on the local tree density (Table 1). We sampled all trees with a diameter >5 cm at the breast height, which potentially reproduced, within widths 10 m from both sides of a route except for topographically inaccessible sides. We recorded the locations of sampled trees using GPSMAP 64 (Garmin) and confirmed them using a fine‐scale topographical map. The tree locations were translated to longitudinal and latitudinal coordinates (m) in the UTM grid zones 53 and 54 and elevations (m) above sea level. To evaluate the density and aggregation of sampled trees, we measured intervals (m) of neighboring trees along a route and obtained the mean and standard deviation (*SD*) of the intervals as well as the coefficient of variation (CV = *SD*/mean) in each population (Table 1). We collected leaves from sampled trees and dried them with silica gel.

**Table 1:** Properties of tree populations of wild cherry species

Species	Population	Site	Bear	Route length (m)	Tree interval		Nuclear genotype			Chloroplast haplotype	
					Mean (m)	CV	*N*	*H*_E_	*F*_IS_	*N*	*H*_E_
*Cerasus jamasakura*	CjAm	Amagi	Absent	5,529	108	1.21	44	0.695	0.038	52	0.757
	CjOk	Okutama	Present	7,859	157	1.23	51	0.705	0.001	52	0.719
	CjKn	Kanazawa	Present	1,361	54	1.22	25	0.694	−0.008	26	0.708
	CjNt	Noto	Absent	2,465	176	0.63	13	0.675	0.051	15	0.257
*Cerasus leveilleana*	CvAs	Ashio	Present	3,745	96	1.82	39	0.741	0.036	40	0.724
	CvOg	Ogawa	Absent	12,801	158	1.58	90	0.737	−0.010	83	0.696
	CvOk	Okutama	Present	7,555	244	1.17	33	0.741	**0.035**	33	0.576
	CvNt	Noto	Absent	4,317	105	1.27	42	0.729	0.001	42	0.382
	CvWk	Wakasa	Present	2,294	143	0.97	17	0.692	−0.033	17	0.794
	CvHr	Hiruzen	Absent	4,559	198	1.03	24	0.738	−0.012	24	0.594
*Padus grayana*	PgAs	Ashio	Present	3,344	59	1.68	53	0.538	0.030	58	0.192
	PgOg	Ogawa	Absent	6,162	101	1.29	56	0.511	0.058	62	0.570
	PgOk	Okutama	Present	4,958	103	2.13	48	0.493	**0.044**	49	0.187
	PgKn	Kanazawa	Present	1,540	42	1.14	36	0.493	0.063	38	0.751
	PgNt	Noto	Absent	3,221	140	1.22	19	0.497	0.032	24	0.761
	PgWk	Wakasa	Present	2,809	85	1.04	29	0.529	−0.032	34	0.665
	PgHr	Hiruzen	Absent	3,823	112	1.26	35	0.485	−0.061	35	0.388

Note

Total length of sampling routes, mean tree interval and coefficient of variation (CV) in tree intervals along a route are shown. Number of sampled trees (*N*), expected heterozygosity (*H*_E_), and fixation index (*F*_IS_) are shown for nuclear genotypes; and *N* and *H*_E_ are shown for chloroplast haplotypes. Bold‐faced values of *F*_IS_ are significantly positive.

### 2.2 Genotyping microsatellites

DNA was extracted from collected leaves in accordance with the standard method (Murray & Thompson, 1980). We determined diploid genotypes of the DNA samples at 17 loci for *C. jamasakura*, 15 loci for *C. leveilleana*, and 14 loci for *P. grayana* in nuclear microsatellites (Table S1). These loci were AM287648, DN556408, DW358868, and DY640849 (Kato et al., 2012; Tsuda, Ueno, et al., 2009); BPPCT005, BPPCT012, BPPCT014, BPPCT026, BPPCT028, BPPCT034, BPPCT037, BPPCT040, and BPPCT041 (Dirlewanger et al., 2002); UDP96‐001, UDP96‐008, UDP96‐018, UDP97‐401, UDP97‐402, UDP98‐024, UDP98‐405, and UDP98‐412 (Testolin et al., 2000); pchcms5 (Sosinski et al., 2000); ASSR17 (Xu, Ma, Xie, Liu, & Cao, 2004) and PMS3 (Cantini, Iezzoni, Lamboy, Boritzki, & Struss, 2001); and M4c, M7a, and MA020a (Yamamoto et al., 2002). We also determined haploid genotypes of the DNA samples at five loci for *C. jamasakura* and *C. leveilleana* and at three loci for *P. grayana* in chloroplast microsatellites (Table S1). These loci were atpFintron_415, atpFintron_628, atpFintron_715, atpFintron_928, and rpL20‐rpS12_248 (S. Kato, A. Matsumoto, R. Mizusawa, Y. Tsuda, & H. Yoshimaru, unpublished). The procedure for determining genotypes followed that described by Kato et al. (2012).

Because *Cerasus* species sometimes hybridize with each other, we identified *C. jamasakura* and *C. leveilleana* based on multilocus genotypes of the nuclear microsatellites using the reference genotypes of identified specimens of these species (Kato et al., 2014). To confirm whether loci were independent with rare null alleles in the nuclear microsatellites, significant deviation from the Hardy–Weinberg equilibrium at every locus and significant linkage disequilibrium for every locus pair were tested in each population using GenePop 4.6.9 (Rousset, 2008) in R 3.3.2 (R Core Team, 2016). For diploid genotypes at the confirmed nuclear loci, the expected heterozygosity (*H*_E_) in each population and the Wright fixation indices within populations (*F*_IS_) and among populations within species (*F*_ST_) were estimated using GenePop 4.6.9. Significant deviation from the Hardy–Weinberg equilibrium (significantly positive or negative *F*_IS_) at all loci in each population was tested using GenePop 4.6.9. Statistical significance (*p* < .05) was adjusted in multiple tests for the loci, locus pairs, and populations in Bonferroni method using the function p.adjust in R 3.3.2.

Because of the complete linkage among loci in a chloroplast haploid genome, we discriminated chloroplast haplotypes based on allelic combinations of the microsatellite loci. For the discriminated chloroplast haplotypes, *H*_E_ in each population and *F*_ST_ among populations within species were also estimated using GenePop 4.6.9.

### 2.3 Genetic differentiation between populations

Pairwise *F*_ST_ of 6, 15, and 21 population pairs in *C. jamasakura*, *C. leveilleana*, and *P. grayana*, respectively, was calculated in nuclear genotypes *F*_ST(b)_ and chloroplast haplotypes *F*_ST(m)_ using GenePop 4.6.9. Spatial distance (m) of these population pairs was calculated from the longitudinal and latitudinal coordinates of the study sites. Interpopulation genetic differentiation parameters in nuclear genotypes *F*_ST(b)_/(1 + *F*_IS_)(1 − *F*_ST(b)_) and chloroplast haplotypes *F*_ST(m)_/(1 − *F*_ST(m)_) were obtained for every population pair in each species (Appendix S1). Relationship between these parameters and the logarithmic spatial distance is thought to be linear on a two‐dimensional ground surface (Rousset, 1997).

The ratio of the genetic differentiation parameters in nuclear genotypes and chloroplast haplotypes depends on the ratio of pollen flow to seed flow between populations in the assumptions of Wright island model (Ennos, 1994; Hamilton & Miller, 2002; Petit et al., 2005; Wright, 1951). The ratio of *F*_ST(m)_/(1 − *F*_ST(m)_) to *F*_ST(b)_/(1 + *F*_IS_)(1 − *F*_ST(b)_) is expected to be 3 when pollen flow is equal to seed flow and to increase as the pollen‐to‐seed‐flow ratio increases (Appendix S1). To verify these patterns, we performed Wilcoxon signed rank test that compared *F*_ST(m)_/(1 − *F*_ST(m)_) with *F*_ST(b)_/(1 + *F*_IS_)(1 − *F*_ST(b)_) and three times of it, using the function wilcox.test (paired = TRUE) in R 3.3.2. To compare the genetic differentiation parameters between the genera, we performed Wilcoxon signed rank test for 18 pairs of *Cerasus* and *Padus* populations at the same combinations of sites where both populations existed.

### 2.4 Spatial genetic structure within populations

The Loiselle kinship coefficients in nuclear genotypes and chloroplast haplotypes were calculated for every pair of sampled trees in each population using SPAGeDi 1.5 (Hardy & Vekemans, 2002). For the tree pairs, three‐dimensional Euclidean distance (m) was calculated from the longitudinal and latitudinal coordinates and the elevations of sampled trees. The spatial distance between trees was transformed to the natural logarithm because relationship between the kinship coefficient and the logarithmic spatial distance is thought to be linear on a two‐dimensional ground surface (Vekemans & Hardy, 2004). In each population, an intercept and a slope of linear regression from the logarithmic spatial distance to the kinship coefficient were estimated from nuclear genotypes and chloroplast haplotypes using the function lm in R 3.3.2. In the regression, we assumed that the kinship coefficient followed a normal distribution, which seemed valid in our samples although a few tree pairs sharing rare haplotypes showed extremely high kinship coefficient in chloroplast haplotypes (Figures S3 and S4). To compare the SGS intensity among populations, the index *Sp* was calculated as *Sp* = −*b*/(1 − *F*_1_), where *b* is the regression slope, and *F*_1_ is the mean kinship coefficient in the first distance class, which was defined as a <10 percentile of spatial distance in each population (Vekemans & Hardy, 2004).

To obtain statistical errors of the indices *F*_1_ and *Sp* and the intercept and slope of regression, Mantel tests were performed. We randomly permuted sampled trees in each population, calculated the pairwise spatial distance, and estimated an intercept and a slope of linear regression from the permuted spatial distance to the observed kinship coefficient. We repeated this procedure 10^4^ times and obtained 2.5 and 97.5 percentiles for each set of the values generated from the permutations using R 3.3.2. When the values estimated from the observed data were lower than the 2.5 percentile or higher than the 97.5 percentile of the permutation values, the observed values were significantly negative or positive, respectively.

The ratio of the regression slopes in nuclear genotypes *b*_b_ and chloroplast haplotypes *b*_m_ is thought to depend on the ratio of pollen dispersal area to seed dispersal area (Chybicki et al., 2016; Hardy & Vekemans, 1999). The ratio of *b*_m_ to *b*_b_(1 + *F*_IS_) is expected to be 3 when pollen dispersal area is equal to seed dispersal area and to increase as the ratio of pollen dispersal area to seed dispersal area increases (Appendix S1). To verify these patterns, we performed Wilcoxon signed rank test that compared *b*_m_ with *b*_b_(1 + *F*_IS_) or three times of it, using the function wilcox.test (paired = TRUE) in R 3.3.2. We calculated the slope ratio *b*_m_/*b*_b_(1 + *F*_IS_) in each population when *b*_b_ < 0.

We compared the indices, *Sp* in nuclear genotypes, *Sp* in chloroplast haplotypes, and the slope ratio *b*_m_/*b*_b_(1 + *F*_IS_), among the 17 populations under different conditions of tree distribution and seed dispersal. We performed Kruskal–Wallis rank sum tests for differences in these indices between *Cerasus* and *Padus* populations and between populations at bear‐absent and bear‐present sites using the function kruskal.test in R 3.3.2. We tested their Kendall rank correlations or their Pearson correlations with the mean tree interval (m) and with the coefficient of variation (CV) in tree intervals using the function cor.test (method = “kendall” or “pearson”) in R 3.3.2. In addition, we selected linear models to predict the three indices, nuclear *Sp*, chloroplast *Sp*, and the slope ratio. These models included possible combinations of four effects of the genus (*Cerasus* = 0 and *Padus* = 1), the mean tree interval, CV in tree intervals, and the bear absence (present = 0 and absent = 1). We selected models with low Akaike Information Criterion (AIC), that is, delta AIC < 2, using the function glm(family = gaussian) and dredge(rank = “AIC”) in R 3.3.2.

## 3 RESULTS

In 17 tree populations of three wild cherry species, the mean interval of neighboring trees along a sampling route ranged from 42 to 244 m, indicating that tree density varied among the populations (Table 1). The standard deviation of tree intervals ranged from 48 to 286 m, and the coefficient of variation (CV) in tree intervals ranged from 0.63 to 2.13, indicating that tree aggregation levels also varied (Table 1). The mean tree interval was significantly larger in *Cerasus* than in *Padus* (*p* = .040; Table 1), indicating higher tree density in *Padus* populations. CV in tree intervals did not significantly differ between the genera (*p* = .329; Table 1). In the 17 populations, nuclear genotypes of 655 sampled trees and chloroplast haplotypes of 685 sampled trees were determined, and the sample sizes of individual populations ranged from 13 to 90 (Table 1).

In the nuclear genotypes, significant deviation from the Hardy–Weinberg equilibrium was detected only at locus BPPCT028 in *C. leveilleana* population and locus UDP97‐402 in *P. grayana* population at Okutama site (*p* < .001) from 17 loci in four *C. jamasakura* populations, 15 loci in six *C. leveilleana* populations, and 14 loci in seven *P. grayana* populations. From 544 locus pairs in *C. jamasakura* populations, 630 locus pairs in *C. leveilleana* populations, and 637 locus pairs in *P. grayana* populations, significant linkage disequilibrium was detected only at locus pair UDP98‐024 and UDP98‐412 in three *C. leveilleana* populations and locus pair BPPCT012 and BPPCT041 in three *P. grayana* populations (*p* < .001). Thus, almost all of the nuclear loci were independent of each other and had rare null alleles. The expected heterozygosity (*H*_E_) was higher (0.692 ≤ *H*_E_ ≤ 0.741) in *Cerasus* populations and lower (0.485 ≤ *H*_E_ ≤ 0.538) in *Padus* populations (Table 1). The fixation index within populations (*F*_IS_) ranged from −0.061 to 0.063, and significantly positive *F*_IS_ was detected in *C. leveilleana* and *P. grayana* populations at Okutama site (*p* < .002; Table 1). The fixation index among populations within species (*F*_ST_) was lowest in *C. jamasakura* (*F*_ST_ = 0.024), intermediate in *C. leveilleana* (*F*_ST_ = 0.046), and highest in *P. grayana* (*F*_ST_ = 0.147).

The observed number of chloroplast haplotypes was 11 in *C. jamasakura* populations, 16 in *C. leveilleana* populations, and 15 in *P. grayana* populations. These chloroplast haplotypes were polymorphic in every population, and their *H*_E_ was comparable (0.187 ≤ *H*_E_ ≤ 0.794) to that of nuclear genotypes (Table 1). *F*_ST_ was higher in chloroplast haplotypes than in nuclear genotypes and showed the same trend among the species, being lowest in *C. jamasakura* (*F*_ST_ = 0.054), intermediate in *C. leveilleana* (*F*_ST_ = 0.168), and highest in *P. grayana* (*F*_ST_ = 0.455).

**Figure 2:**
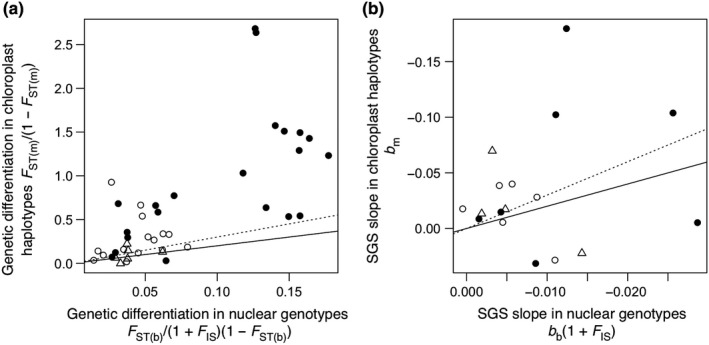
(a) Genetic differentiation between populations in nuclear genotypes, which is shown as *F*_ST_/(1 + *F*_IS_)(1 – *F*_ST_), and chloroplast haplotypes, which is shown as *F*_ST_/ (1 – *F*_ST_), where *F*_IS_ and *F*_ST_ are Wright fixation indices within and between populations, respectively. (b) SGS intensity within populations in nuclear genotypes and chloroplast haplotypes, which is shown as slope of regression of Loiselle kinship coefficient on natural logarithm of spatial distance (m). The slopes are *b*_b_ and *b*_m_ in nuclear genotypes and chloroplast haplotypes, respectively. The slope in nuclear genotypes is shown as *b*_b_(1 + *F*_IS_). The axes of b are inverted to compare plots a and b. Symbols indicate *Cerasus jamasakura* (triangle), *C. leveilleana* (open circles), and *Padus grayana* (filled circles). Solid and dotted lines indicate *y* = 2*x* and *y* = 3*x*, where pollen flow (a) or pollen dispersal area (b) is positive and equal to seed flow or seed dispersal area, respectively

The interpopulation genetic differentiation parameters in nuclear genotypes *F*_ST(b)_/(1 + *F*_IS_)(1 − *F*_ST(b)_) and chloroplast haplotypes *F*_ST(m)_/(1 − *F*_ST(m)_) were obtained from pairwise *F*_ST_ of 42 population pairs in the three species (Appendix S1, Figure 2a). Linear regression from the logarithmic geographic distance to the genetic differentiation parameters between populations varied among the species (Figure S2). The linear regression fitted well to the population pairs except for nuclear genotypes in *P. grayana*, which showed the extensive divergence of the three eastern populations at Ashio, Ogawa, and Okutama sites from the others (Figure S2). The regression slopes in both nuclear genotypes and chloroplast haplotypes were steepest in *P. grayana*, followed by *C. leveilleana* and *C. jamasakura* (Figure S2). In 18 pairs of *Cerasus* and *Padus* populations at the same combinations of sites where both populations existed, the genetic differentiation parameters were significantly higher in *Padus* than in *Cerasus* in nuclear genotypes (*p* < .001) and in chloroplast haplotypes (*p* = .002; Figure 2a). The genetic differentiation parameter in chloroplast haplotypes was significantly higher and significantly >3 times higher than that in nuclear genotypes (*p* < .001), indicating that pollen flow exceeded seed flow between populations (Appendix S1). The ratio of the genetic differentiation parameter in chloroplast haplotypes to that in nuclear genotypes was significantly higher in *Padus* (mean = 9.35) than in *Cerasus* (5.92, *p* = .008; Figure 2a), indicating more extensive pollen flow relative to seed flow in *Padus* (pollen‐to‐seed‐flow ratio = 7.35) than in *Cerasus* (3.92).

In the 17 populations, linear regression from the logarithmic spatial distance to the kinship coefficient was estimated in nuclear genotypes (Figure S3) and chloroplast haplotypes (Figure S4). The regression slopes in nuclear genotypes *b*_b_ and chloroplast haplotypes *b*_m_ were obtained, and *b*_b_(1 + *F*_IS_) and *b*_m_ were examined to evaluate pollen and seed dispersal (Appendix S1). In nuclear genotypes, significantly negative slopes *b*_b_(1 + *F*_IS_) and significantly positive regression intercepts and indices *F*_1_ and *Sp* were detected in 10 populations (*p* < .05; Table 2). In chloroplast haplotypes, this pattern was detected in five populations although an inverse pattern with a significantly positive slope *b*_m_ and significantly negative intercept and *Sp* was detected in *P. grayana* population at Hiruzen site (*p* < .05; Table 2). The slopes did not significantly differ between *Cerasus* and *Padus* populations in chloroplast haplotypes (*p* = .696) or nuclear genotypes (*p* = .143) although much steeper slopes were found only in *Padus* (Figure 2b). The slope in chloroplast haplotypes *b*_m_ was neither significantly >3 times higher than that in nuclear genotypes *b*_b_(1 + *F*_IS_) (*p* = .459) nor significantly higher than it (*p* = .132; Figure 2b). The mean slope ratio *b*_m_/*b*_b_(1 + *F*_IS_) in 16 populations with *b*_b_ < 0 was 5.17, while the ratio varied from –3.67 to 21.88 among the populations (Table 2).

**Table 2:** Indices of fine‐scale spatial genetic structure in nuclear genotypes and chloroplast haplotypes in tree populations of wild cherry species

Population	Nuclear genotype (10^3^)				Chloroplast haplotype (10^3^)				Slope ratio
	*F*_1_	*Sp*	Intercept	Slope *b*_b_(1 + *F*_IS_)	*F*_1_	*Sp*	Intercept	Slope *b*_m_	*b*_m_/*b*_b_(1 + *F*_IS_)
CjAm	1.65	3.08	19.90	−3.19	**179.69**	**85.03**	**463.90**	**−69.75**	21.88
CjOk	**13.26**	**4.87**	**35.62**	**−4.81**	80.74	18.71	126.89	−17.20	3.58
CjKn	7.31	1.90	10.22	−1.87	98.50	14.74	71.17	−13.29	7.11
CjNt	12.27	13.79	85.43	−14.32	−44.73	−21.29	−142.56	22.24	−1.55
CvAs	**15.28**	**8.56**	**47.96**	**−8.73**	**172.45**	34.00	163.41	−28.14	3.22
CvOg	**17.05**	**5.81**	**41.59**	**−5.65**	**113.78**	**45.07**	**291.74**	**−39.94**	7.07
CvOk	**17.23**	**4.41**	**32.01**	**−4.49**	20.51	5.61	41.23	−5.50	1.22
CvNt	**19.62**	**11.18**	**70.15**	**−10.97**	11.18	−28.78	−182.28	28.46	−2.59
CvWk	17.95	−0.51	−2.10	0.49	158.73	20.96	108.62	−17.64	NA
CvHr	−13.26	4.06	25.57	−4.06	31.28	39.83	237.48	−38.58	9.50
PgAs	**77.95**	**30.19**	**157.24**	**−28.67**	13.93	5.31	29.94	−5.23	0.18
PgOg	**42.43**	**12.24**	**79.48**	**−12.40**	**371.35**	**285.84**	**1,228.70**	**−179.69**	14.49
PgOk	**29.09**	**10.92**	**67.00**	**−11.07**	**653.54**	**295.12**	**651.23**	**−102.25**	9.24
PgKn	−3.77	1.43	6.85	−1.53	5.21	8.58	45.65	−8.53	5.58
PgNt	**73.22**	**26.80**	**159.55**	**−25.63**	**225.11**	**134.01**	**665.65**	**−103.84**	4.05
PgWk	1.69	4.41	27.81	−4.26	21.79	15.07	91.07	−14.74	3.46
PgHr	**22.98**	**9.35**	**56.17**	**−8.57**	−64.02	**−29.58**	**−190.49**	**31.47**	−3.67
Mean	20.56	8.95	54.02	−8.82	132.07	59.86	243.24	−37.10	5.17
*SD*	24.27	8.42	46.66	8.05	175.61	97.34	361.44	54.26	6.55

Note

*F*_1_ is Loiselle kinship coefficient in the first distance class (<10 percentile). *Sp* is an index of SGS intensity. Intercept and slope are obtained from regression of the kinship coefficient on natural logarithm of spatial distance (m). The slopes are *b*_b_ and *b*_m_ in nuclear genotypes and chloroplast haplotypes, respectively. The slope in nuclear genotypes is shown as *b*_b_(1 + *F*_IS_). Slope ratio is *b*_m_/*b*_b_(1 + *F*_IS_) when *b*_b_ is negative (NA because of *b*_b_ ≥ 0). Bold‐faced values are significantly positive or negative. Mean and standard deviation (*SD*) of the values in the populations are shown.

**Figure 3:**
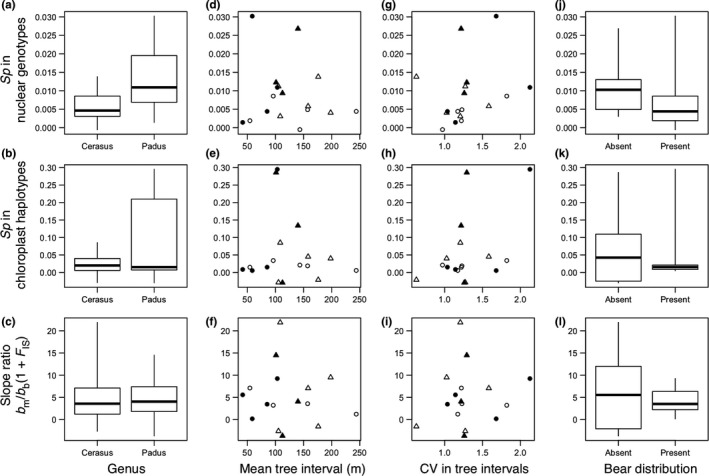
Comparison of SGS indices in populations of different genera (*Cerasus* and *Padus*, a–c), with local tree densities (mean tree interval, d–f) and tree aggregation levels (coefficient of variation in tree intervals, g–i), and at bear‐absent and bear‐present sites (j–l). The SGS indices are *Sp* in nuclear genotypes (a, d, g, j) and *Sp* in chloroplast haplotypes (b, e, h, k) and ratio of regression slope in chloroplast haplotypes to that in nuclear genotypes when the latter is negative (shown as *b*_m_/*b*_b_(1 + *F*_IS_); c, f, i, l). Lines indicate medians, boxes indicate 25 and 75 percentiles, and whiskers indicate ranges (a–c, j–l). Open and filled symbols indicate *Cerasus* and *Padus* populations, respectively (d–i). Triangles and circles indicate bear‐absent and bear‐present sites, respectively (d–i)

Three indices, *Sp* in nuclear genotypes, *Sp* in chloroplast haplotypes, and the slope ratio *b*_m_/*b*_b_(1 + *F*_IS_), did not significantly differ between *Cerasus* and *Padus* populations (*p* > .143) although *Sp* tended to be higher in *Padus* than in *Cerasus* (Figure 3a–c). These indices were not significantly correlated with the mean tree interval (*p* > .825; Figure 3d–f). The correlations between CV in tree intervals and *Sp* were significant in nuclear genotypes (Kendall *τ* = .397, *p* = .027) and marginally significant in chloroplast haplotypes (Pearson *r* = .472, *p* = .055), indicating more intensive SGS as trees became more aggregated (Figure 3g,h). The slope ratio was not significantly correlated with CV in tree intervals (*p* = 1.000; Figure 2i). The absence of Asian black bears did not significantly affect any indices (*p* > .149; Figure 3j–l). The model selection was consistent with these comparisons. Models selected to predict nuclear *Sp* frequently included a positive effect of the genus *Padus*, and models selected to predict chloroplast *Sp* frequently included positive effects of the genus *Padus* and CV in tree intervals (Table S2). A model selected to predict the slope ratio with the lowest AIC included no effects (Table S2).

## 4 DISCUSSION

Maternally inherited loci are predicted to have higher interpopulation genetic differentiation and more intensive within‐population SGS than biparentally inherited loci because of smaller effective population sizes and fewer opportunities of gene dispersal in the former loci (Chybicki et al., 2016; Ennos, 1994; Hamilton & Miller, 2002; Hardy & Vekemans, 1999; Petit et al., 2005). To compare these loci, we examined chloroplast haplotypes in maternally inherited loci and nuclear genotypes in biparentally inherited loci. As predicted, the estimated genetic differentiation between populations of the studied cherry species was higher in chloroplast haplotypes than in nuclear genotypes. The ratio of interpopulation genetic differentiation in maternally and biparentally inherited loci is thought to depend on the ratio of pollen and seed flow between populations (Ennos, 1994). The estimated pollen‐to‐seed‐flow ratio (3.92 in *Cerasus* and 7.35 in *Padus*) indicates that pollen flow exceeds seed flow. This ratio is less than the median ratio (17) in various plant taxa (Petit et al., 2005). Among these plant taxa, insect‐pollinated and fleshy‐fruited species, for which pollen dispersal is relatively limited while frugivorous vertebrates render long‐distance seed dispersal, tend to show low (<5) pollen‐to‐seed‐flow ratios (Oddou‐Muratorio, Petit, Le Guerroue, Guesnet, & Demesure, 2001; Petit et al., 2005). Thus, the studied cherry species are typical examples of this plant category.

In contrast to the interpopulation genetic differentiation, the estimated within‐population SGS intensity did not significantly differ between nuclear genotypes and chloroplast haplotypes due to a large variation among populations. This variation in the SGS intensity may result from ecological variabilities among populations, such as colonization history, although small sample sizes in the within‐population SGS analyses are partially responsible for the nonsignificant results. Fine‐scale SGS is likely to differ between the stages of population development from initial colonization of open habitats to subsequent regeneration in the colonized habitats (Hamrick & Trapnell, 2011). The SGS intensity seems low when founders from different maternal sources independently colonize an open habitat. The SGS intensity in the colonized habitat seems to increase as descendants from the founders regenerate around them. In contrast, kin‐structured seed dispersal, which leads to aggregated colonization from the same maternal source, seems to create intensive SGS in a founder population (García & Grivet, 2011; Torimaru, Tani, Tsumura, Nishimura, & Tomaru, 2007). Regeneration of the founders with overlapping seed shadows may reduce the SGS intensity. Subsequent immigration to the colonized habitat would alter the SGS intensity (Hamrick & Trapnell, 2011). Thus, the SGS intensity varies depending on the stages of colonization history, which seems specific to individual populations. A significantly negative *Sp* index, which was not expected from the equilibrium of spatial population dynamics (Furstenau & Cartwright, 2016), was observed in a population. This result may reflect a nonequilibrium state in population development. Therefore, the variation in fine‐scale SGS in the studied cherry populations may result from population‐specific states in colonization history.

Consistent differences in SGS within and between populations were found between *Cerasus* and *Padus*. In both spatial scales, more intensive SGS was observed in *Padus* than in *Cerasus*. These findings suggest lower effective population size and/or more limited gene flow in *Padus* than in *Cerasus* (Ennos, 1994; Hardy & Vekemans, 1999). Furthermore, the estimated pollen‐to‐seed‐flow ratio was higher in *Padus* than in *Cerasus*, suggesting that the intensive SGS mainly results from more limited seed flow. Because the breeding systems and the pollen and seed dispersal systems are similar between these genera, their differences in SGS are related to other life history traits. A candidate trait is regeneration strategy, which is associated with different patterns of spatial tree distribution. At the study sites, local tree density was higher in *Padus* than in *Cerasus*. Also in a sparse pine forest, the germination rate of sound seeds was higher in *P. grayana* than in *C. leveilleana*, resulting in higher juvenile density (Shirota, Miyauchi, Saito, Maruyama, & Okano, 2015a, 2015b). In a mature broadleaf forest, *P. grayana* occurred in only young stands, whereas *C. leveilleana* occurred in both young and old stands (Masaki, 2002). These features indicate denser and more aggregated trees in more restricted regenerating habitats in *Padus* than in *Cerasus*. In tropical forests, more intensive SGS was observed in tree species, of which local density was higher (Hardy et al., 2006). Denser and more aggregated distribution may result from shorter seed dispersal and may result in shorter pollen dispersal, leading to more intensive SGS (Doligez, Baril, & Joly, 1998). However, higher local density may also result in larger effective population size, which leads to less intensive SGS (Hardy & Vekemans, 1999). Thus, because effects of spatial distribution on SGS are complicated (Doligez et al., 1998), it is difficult to determine the factors responsible for the differences in SGS between *Cerasus* and *Padus*.

In the studied cherry populations, the SGS intensity increased as trees were more aggregated but did not depend on local tree density. As discussed above, aggregation is expected to increase the SGS intensity (Doligez et al., 1998), which is consistent with the former result. However, relatives are not always aggregated, and clumping of seedlings from various maternal sources can reduce the SGS intensity (Sagnard, Oddou‐Muratorio, Pichot, Vendramin, & Fady, 2011). Therefore, the aggregation observed in the studied cherry trees often consists of siblings originating from the same maternal source. As mentioned previously, local density has the opposite effects on the SGS intensity, which increases due to the bias of gene dispersal toward shorter (Hardy et al., 2006) or decreases because the effective population density increases (Hardy & Vekemans, 1999). In an herb species in segregated pastures, the SGS intensity decreased as the population size in individual pastures increased (Rico & Wagner, 2016). In the studied cherry populations, the opposite effects may counterbalance each other, resulting in the unclear effect of local tree density on the SGS intensity.

The loss of effective seed dispersers, Asian black bears, is expected to reduce seed dispersal distance and result in more intensive SGS in chloroplast haplotypes than in nuclear genotypes (Calvino‐Cancela et al., 2012; Gelmi‐Candusso, Heymann, & Heer, 2017; Silvestrini et al., 2015). However, the ratio of regression slopes in chloroplast haplotypes and nuclear genotypes did not differ between populations at bear‐absent and bear‐present sites. Not only bears but also other animals, such as birds, macaques, and carnivores disperse seeds of the studied cherry species (Fujitsu et al., 2016; Koike, Kasai, et al., 2008; Koike, Morimoto, et al., 2008; Masaki et al., 2012). These animals, which are common in both bear‐absent and bear‐present sites, contribute to seed dispersal to some extent. This contribution may reduce the impacts of bear loss on fine‐scale SGS. In addition to the mitigation of bear loss, the duration of bear loss at bear‐absent sites seems insufficient to increase the SGS intensity because the formation of fine‐scale SGS requires several generations and subsequent replacement of trees (Hamilton & Miller, 2002), which may spend some hundred years in the studied cherry species.

Comparison of SGS in multiple spatial scales using genetic markers with different modes of inheritance is a feasible indirect method to evaluate pollen and seed dispersal (Chybicki et al., 2016; Hamrick & Trapnell, 2011; Petit et al., 2005). The findings from replicated populations of wild cherry trees imply that SGS is more variable in smaller spatial scales. Thus, the precise evaluation of gene dispersal seems to require a proper spatial scale, at which signals of the focal dispersal process exceed noises in SGS. Furthermore, the findings suggest that various ecological factors in local populations affect fine‐scale SGS. Because of these potential factors, it is difficult to detect the impacts of a specific factor on fine‐scale SGS. In spite of these difficulties, fine‐scale SGS gives insight into the characteristics of gene dispersal and the maintenance mechanisms of genetic diversity in local plant populations.

## CONFLICT OF INTEREST

None declared.

## AUTHOR CONTRIBUTIONS

SKo, TM, and TN designed this study. KS, SKi, SN, and TN conducted the field work. KS, SKi, and TN arranged the genetic data. TN analyzed the data and wrote the manuscript.

## Data Availability

Locations, tree sizes, nuclear multilocus genotypes, and chloroplast haplotypes of sampled trees in populations of cherry species in study sites: Dryad https://doi.org/10.5061/dryad.gv5qp70.
