# Supplementary material for: Multiscale spatial genetic structure within and between populations of wild cherry trees in nuclear genotypes and chloroplast haplotypes
Source: Ecol Evol. 2019 Sep 4;9(19):11266–76. doi: 10.1002/ece3.5628 (PMC6802027; doi:10.1002/ece3.5628)
Supplement: Supplementary file 1 [file ECE3-9-11266-s001.pdf]

Supporting Information

Multiscale spatial genetic structure within and between populations of wild cherry trees in nuclear genotypes and chloroplast haplotypes

Teruyoshi Nagamitsu, Kato Shuri, Satoshi Kikuchi, Shinsuke Koike, Shoji Naoe, and Takashi Masaki

This pdf file includes Appendix S1, Tables S1 and S2, and Figures S1, S2, S3, and S4.

**Appendix S1**

The Wright's fixation index $F_{ST}$ is an index to evaluate genetic differentiaiton between diploid populations. Wright (1951) provided the equation

$$F_{ST} = \frac{1}{\frac{4Nm}{1+F}+1} \qquad [1]$$

where $N$ is the effective population size, $m$ is the migration rate between populations, and $F$ is the inbreeding coefficient (same as $F_{IS}$ of the Wright's fixation index in a population). This equation is valid in the Wright's island model with the assumptions: (1) no mutation and no selection; (2) equal number of migrants between populations in both directions; (3) identical male and female effective population sizes in both populations; and (4) equilibrium between genetic drift and gene flow. Thus, the genetic differentiation increases as the effective population size decreases and/or as gene flow between populations decreases.

Ennos (1994) provided the equation that derived the ratio of pollen and seed flow between populations from biparentally and maternally inherited genetic markers. Pollen flow $m_p$ is defined as the number of pollen dispersed between populations per generation, which fetilizes an ovule, resulting in successful seed production. Seed flow $m_s$ is defined as the number of seeds dispersed between populations per generation, which successfully establish in the populations. Genetic differentiation between populations is obtained from not only biparentally inherited genetic markers in a nuclear genome but also maternally inherited genetic markers in an organelle genome. Thus, $F_{ST}$ is obtained from biparentally inherited genetic markers $F_{ST(b)}$ or maternally inherited genetic markers $F_{ST(m)}$. The ratio of pollen and seed flow between populations is

$$\frac{m_p}{m_s} = \frac{(1+F)\left(\frac{1}{F_{ST(b)}}-1\right)-2\left(\frac{1}{F_{ST(m)}}-1\right)}{\left(\frac{1}{F_{ST(m)}}-1\right)} \qquad [2].$$

The equation [2] is shown in the equation 5a of Ennos (1994) and the equation 1 of Petit et al. (2005). When genetic differentiation in biparentally and maternally inherited genetic markers is expressed as parameters

$$a_b = \frac{F_{ST(b)}}{1-F_{ST(b)}} \qquad [3] \text{ and}$$

$$a_m = \frac{F_{ST(m)}}{1-F_{ST(m)}} \qquad [4],$$

the equation [2] is transformed to

$$\frac{m_p}{m_s} = (1 + F)\frac{a_m}{a_b} - 2 \qquad [5],$$

because

$$\frac{1}{F_{ST(b)}} - 1 = \frac{1}{a_b} \qquad [3a] \text{ and}$$

$$\frac{1}{F_{ST(m)}} - 1 = \frac{1}{a_m} \qquad [4a].$$

The equation [5] is transformed to

$$\frac{\frac{a_m}{a_b}}{1+F} = \frac{m_p}{m_s} + 2 \qquad [6].$$

In random mating populations, *F* is 0. Thus, the equation [6] is

$$\frac{a_m}{a_b} = \frac{m_p}{m_s} + 2 \qquad [6a].$$

Threrefore, the ratio of genetic differentiation parameters in maternally and biparentally inherited genetic markers ($a_m/a_b$) simply depends on the ratio of pollen and seed flow between populations ($m_p/m_s$).

The slope of linear regression from the logarithmic distance to the kinship coefficient within populations is an index to evaluate fine-scale spatial genetic structure (SGS). Hardy and Vekemans (1999) provided the equation for the SGS slope

$$b = -\frac{1-F}{(1+(k-1)F)2\pi D_e \sigma^2} \qquad [7],$$

where $D_e$ is the effective population density, $\sigma^2$ is the squared mean distance of gene dispersal (i.e., the area of gene dispersal), and *k* is the ploidy level. The equation [7] is shown in the equation 15 of Hardy and Vekemans (1999). Thus, the SGS slope becomes steeper as the effective population density decreases and/or as the gene dispersal area decreases. In biparentally inherited genetic markers, these parameters are

$$D_e = 4r(1-r)D \quad [8],$$

$$\sigma^2 = \frac{\sigma_p^2}{2} + \sigma_s^2 \quad [9], \text{ and}$$

$$k = 2 \quad [10],$$

where *r* is the proportion of effectively reproducing females, *D* is the population density, $\sigma_p^2$ is the squared mean distance (area) of pollen dispersal, $\sigma_s^2$ is the squared mean distance (area) of seed dispersal (Chybicki et al. 2016). Thus, the SGS slope in biparentally inherited genetic markers is

$$b_b = -\frac{1-F}{(1+F)8\pi r(1-r)D\left(\frac{\sigma_p^2}{2}+\sigma_s^2\right)} \quad [11]$$

In maternally inherited genetic markers, those parameters are

$$D_e = rD \quad [12],$$

$$\sigma^2 = \sigma_s^2 \quad [13], \text{ and}$$

$$k = 1 \quad [14].$$

Thus, the SGS slope in maternally inherited genetic markers is

$$b_m = -\frac{1-F}{2\pi r D\sigma_s^2} \quad [15].$$

From the equations [11] and [15], the ratio of SGS slopes in maternally and biparentally inherited genetic markers is

$$\frac{b_m}{b_b} = 2(1+F)(1-r)\left(\frac{\sigma_p^2}{\sigma_s^2} + 2\right) \quad [16].$$

In hermaphrodites, *r* is 0.5. Thus, the equation [16] is transformed to

$$\frac{b_m}{b_b(1+F)} = \frac{\sigma_p^2}{\sigma_s^2} + 2 \quad [17].$$

In random mating populations, *F* is 0. Thus, the equation [17] is

$$\frac{b_m}{b_b} = \frac{\sigma_p^2}{\sigma_s^2} + 2 \quad [17a].$$

Threrefore, the ratio of SGS slopes in maternally and biparentally inherited genetic

markers ($b_m/b_b$) simply depends on the ratio of pollen and seed dispersal area within populations ($\sigma_p^2/\sigma_s^2$).

According to the equations [6a] and [17a], the genetic differentiation between populations and the SGS slopes within populations are useful to estimate the ratio of pollen and seed dispersal at respective scales in the same manner. In addition, the ratio of both parameters in maternally and biparentally inherited genetic markers ($a_m/a_b$ and $b_m/b_b$) is predicted to be more than 2. When the number of pollen and seed flow between populations are equal ($m_p/m_s = 1$) or when the area of pollen and seed dispersal within populations are equal ($\sigma_p^2/\sigma_s^2 = 1$), the ratio is expected to be 3.

Table S1. Microsatellites in nuclear and chloroplast genomes for studied cherry species. The number of alleles at loci that applied to each species is shown.

| Locus | No. of alleles | | | Size (bp) | Primer sequences ('5–'3) | | Reference |
|---|---|---|---|---|---|---|---|
| | *Cj* | *Cv* | *Pg* | | Forward | Reverse | |
| PMS3 | 21 | 48 | | 150–260 | TGGACTTCACTCATTTCAGAGA | ACTGCAGAGAATTTCACAACCA | Cantini et al. 2001 |
| BPPCT005 | 20 | 30 | | 120–220 | GCTAGCAGGGCACTTGATC | ACGCGTGTACGGTGGAT | Dirlewanger et al. 2002 |
| BPPCT012 | | | 26 | 110–190 | ACTTCCATTGTCAGGCATCA | GGAGCAACGATGGAGTGC | Dirlewanger et al. 2002 |
| BPPCT014 | | 28 | 3 | 160–250 | TTGTCTGCCTCTCATCTTAACC | CATCGCAGAGAACTGAGAGC | Dirlewanger et al. 2002 |
| BPPCT026 | 15 | 20 | | 120–200 | ATACCTTTGCCACTTGCG | TGAGTTGGAAGAAAACGTAACA | Dirlewanger et al. 2002 |
| BPPCT028 | 17 | 19 | | 140–200 | TCAAGTTAGCTGAGGATCGC | GAGCTTGCCTATGAGAAGACC | Dirlewanger et al. 2002 |
| BPPCT034 | 27 | 21 | | 200–310 | CTACCTGAAATAAGCAGAGCCAT | CAATGGAGAATGGGGTGC | Dirlewanger et al. 2002 |
| BPPCT037 | 12 | 10 | | 110–190 | CATGGAAGAGGATCAAGTGC | CTTGAAGGTAGTGCCAAAGC | Dirlewanger et al. 2002 |
| BPPCT040 | | | 2, 3a | 120–140 | ATGAGGACGTGTCTGAATGG | AGCCAAACCCCTCTTATACG | Dirlewanger et al. 2002 |
| BPPCT041 | | | 12 | 200–240 | CAATAAGGCATTTGGAGGC | CAGCCGAACCAAGGAGAC | Dirlewanger et al. 2002 |
| DN556408 | | | 3 | 180–190 | ATTCTTCTTCCGCCACTTCTGAAT | GTTTAGATCACAGCACGCGAAAATG | Kato et al. 2012 |
| pchcms5 | 21 | | | 220–340 | CGCCCATGACAAACTTA | GTCAAGAGGTACACCAG | Sosinski et al. 2000 |
| UDP96-001 | 8 | 8 | | 90–130 | AGTTTGATTTTCTGATGCATCC | TGCCATAAGGACCGGTATGT | Testolin et al. 2000 |
| UDP96-008 | | | 10 | 120–150 | TTGTACACACCCTCAGCCTG | TGCTGAGGTTCAGGTGAGTG | Testolin et al. 2000 |
| UDP96-018 | 11 | | | 250–290 | TTCTAATCTGGGCTATGGCG | GAAGTTCACATTTACGACAGGG | Testolin et al. 2000 |
| UDP97-401 | | | 5 | 110–120 | TAAGAGGATCATTTTTGCCTTG | CCCTGGAGGACTGAGGGT | Testolin et al. 2000 |
| UDP97-402 | | | 23 | 110–170 | TCCCATAACCAAAAAAAACACC | TGGAGAAGGGTGGGTACTTG | Testolin et al. 2000 |
| UDP98-024 | 9 | 8 | | 80–130 | CCTTGATGCATAATCAAACAGC | GGACACACTGGCATGTGAAG | Testolin et al. 2000 |
| UDP98-405 | | | 8 | 100–120 | ACGTGATGAACTGACACCCA | GAGTCTTTGCTCTGCCATCC | Testolin et al. 2000 |
| UDP98-412 | 22 | 20 | 4 | 80–180 | AGGGAAAGTTTCTGCTGCAC | GCTGAAGACGACGATGATGA | Testolin et al. 2000 |
| AM287648 | 10 | 7 | 4 | 260–370 | ATGATGCTACCACAAGGGACTCGT | GTTTAGCTGCACATACGCTTTTACCTCC | Tsuda et al. 2009b |
| DW358868 | 5 | 3 | | 240–280 | ATTGATTTCGGACCCATAAAACCC | GTTTACAACATCACGTACGGGCCTC | Tsuda et al. 2009b |
| DY640849 | 11 | 21 | 7 | 280–340 | ATAGGCCAGGCAAATAGCGAAGTA | GTTTCCTCTGTAGCTCCCAAGTTTTCG | Tsuda et al. 2009b |
| ASSR17 | | | 6 | 150–170 | GGACTGGACTGTGGATTGTTTTTG | AGTCATCCACCGTGCCAGTTTTA | Xu et al. 2004 |
| M4c | 5 | 7 | | 70–120 | GAATTTGTTCTCTCTCTCTC | GGAAGCGTTCGTCTGCAAAT | Yamamoto et al. 2002 |
| M7a | 4 | | | 150–190 | GAAGAAAGACTGAAACAACG | CCAGTTGAGAGTGTCTTTGA | Yamamoto et al. 2002 |
| MA020a | 7 | 13 | | 160–210 | CTTGCCCATTTATGTACTGA | TATATCGCATAATCACGGTC | Yamamoto et al. 2002 |
| atpFintron_415 | 3 | 3 | 2 | 152–155 | TTCCCGAACCAAACATGAAT | TTGGATTAGCGATCCGTTTC | Kato et al. unpublished |
| atpFintron_628 | 5 | 5 | | 192–197 | GAAACGGATCGCTAATCCAA | ACAAATCGGAAAAACGGGTA | Kato et al. unpublished |
| atpFintron_715 | 4 | 3 | | 172–175 | ACCCGTTTTTCCGATTTGTT | GAAGTTCAGATGCAGCATGG | Kato et al. unpublished |
| atpFintron_928 | 3 | 3 | 8 | 212–247 | TTTGAACCCGCTTCCATATT | CATGATTCCGCGAATTTCTT | Kato et al. unpublished |
| rpL20-rpS12_248 | 6 | 6 | 6 | 195–205 | TATAACCTTCCCGACCACGA | AAAGAAGGGCTCCGGTGTAT | Kato et al. unpublished |

a: Two loci are available in PCR products from BPPCT040 primers

Table S2. Selected models including effects of genus, tree distribution, and bear loss to predict SGS indices.

*Sp* in nuclear genotypes

| Intercept | Effect | | | | AIC | Delta AIC | Akaike weight |
|---|---|---|---|---|---|---|---|
| | Genus *Padus* | Mean tree interval (m) | CV in tree intervals | Bear loss | | | |
| 5.71 | 7.91 | NS | NS | NS | -113.6 | 0.00 | 0.230 |
| 7.72 | 8.19 | NS | NS | 4.00 | -112.9 | 0.64 | 0.167 |
| 1.21 | 7.22 | NS | 5.80 | 5.05 | -112.2 | 1.41 | 0.144 |
| 1.24 | 7.24 | NS | 3.69 | NS | -112.1 | 1.51 | 0.108 |
| 2.54 | 9.06 | 0.02 | NS | NS | -111.9 | 1.68 | 0.099 |

*Sp* in chloroplast haplotypes

| Intercept | Effect | | | | AIC | Delta AIC | Akaike weight |
|---|---|---|---|---|---|---|---|
| | Genus *Padus* | Mean tree interval (m) | CV in tree intervals | Bear loss | | | |
| -110.00 | 61.09 | NS | 108.40 | NS | -30.5 | 0.00 | 0.172 |
| -114.80 | NS | NS | 131.70 | NS | -30.3 | 0.20 | 0.156 |
| -110.30 | 60.90 | NS | 127.67 | 46.06 | -29.9 | 0.58 | 0.129 |
| -197.79 | 83.52 | 0.48 | 123.64 | NS | -29.8 | 0.73 | 0.119 |
| -115.10 | NS | NS | 150.98 | 46.31 | -29.5 | 0.95 | 0.107 |
| 21.39 | 80.66 | NS | NS | NS | -29.3 | 1.19 | 0.095 |
| -180.80 | 38.15 | 0.39 | 136.61 | 78.95 | -28.7 | 1.75 | 0.072 |

Slope ratio

| Intercept | Effect | | | | AIC | Delta AIC | Akaike weight |
|---|---|---|---|---|---|---|---|
| | Genus *Padus* | Mean tree interval (m) | CV in tree intervals | Bear loss | | | |
| 5.17 | NS | NS | NS | NS | 108.5 | 0.00 | 0.281 |
| 6.15 | NS | NS | NS | 1.95 | 110.1 | 1.62 | 0.125 |
| 2.19 | NS | NS | 2.29 | NS | 110.3 | 1.76 | 0.117 |
| 6.64 | NS | -0.01 | NS | NS | 110.4 | 1.84 | 0.112 |
| 5.49 | -0.73 | NS | NS | NS | 110.5 | 1.95 | 0.106 |

NS: Not selected

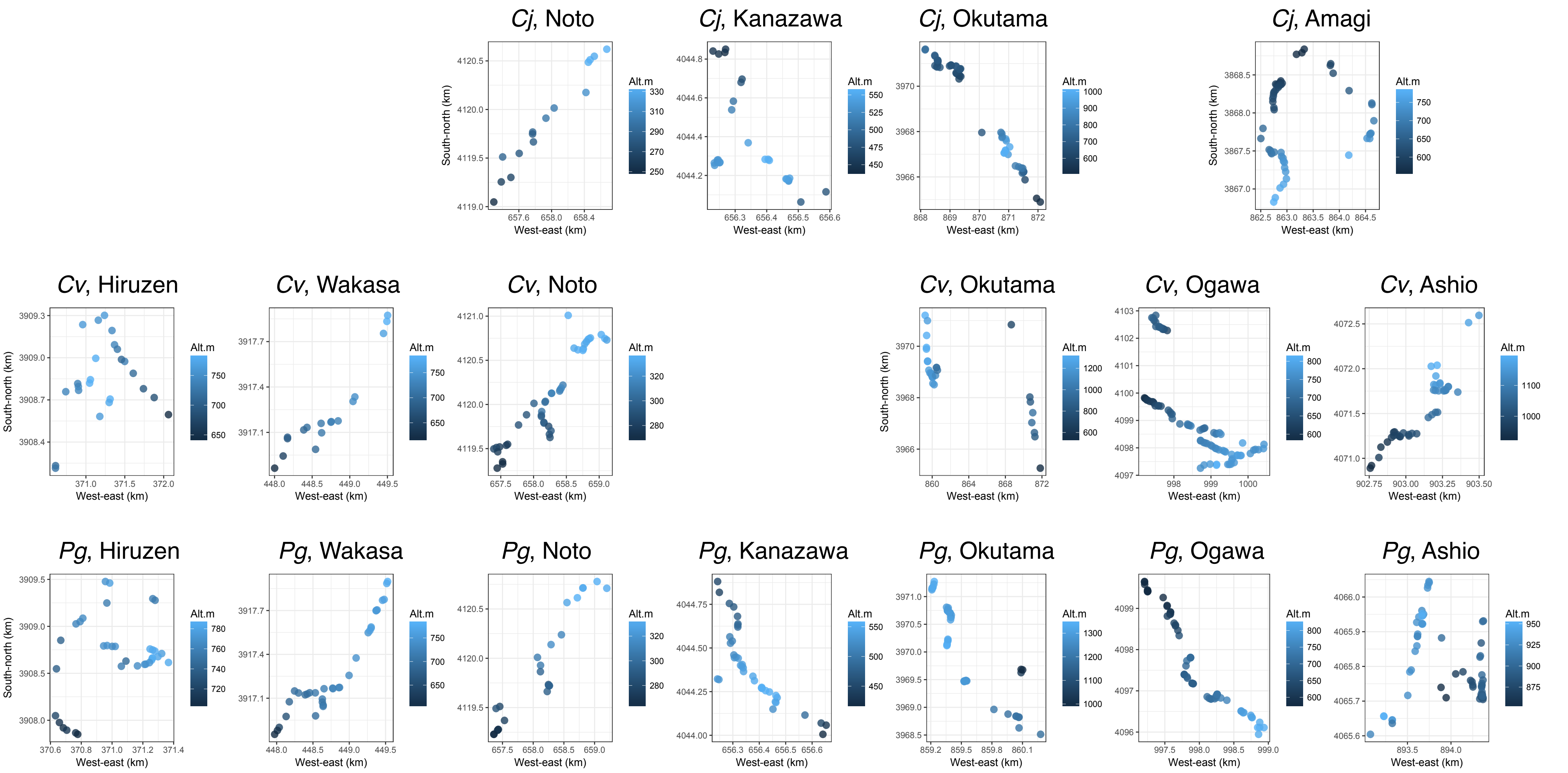


**Fig. S1**. Spatial distribution of sampled trees in populations of cherry species (*Cj*: *C. jamasakura*, *Cv*: *C. leveilleana*, and *Pg*: *P. grayana*) at study sites.

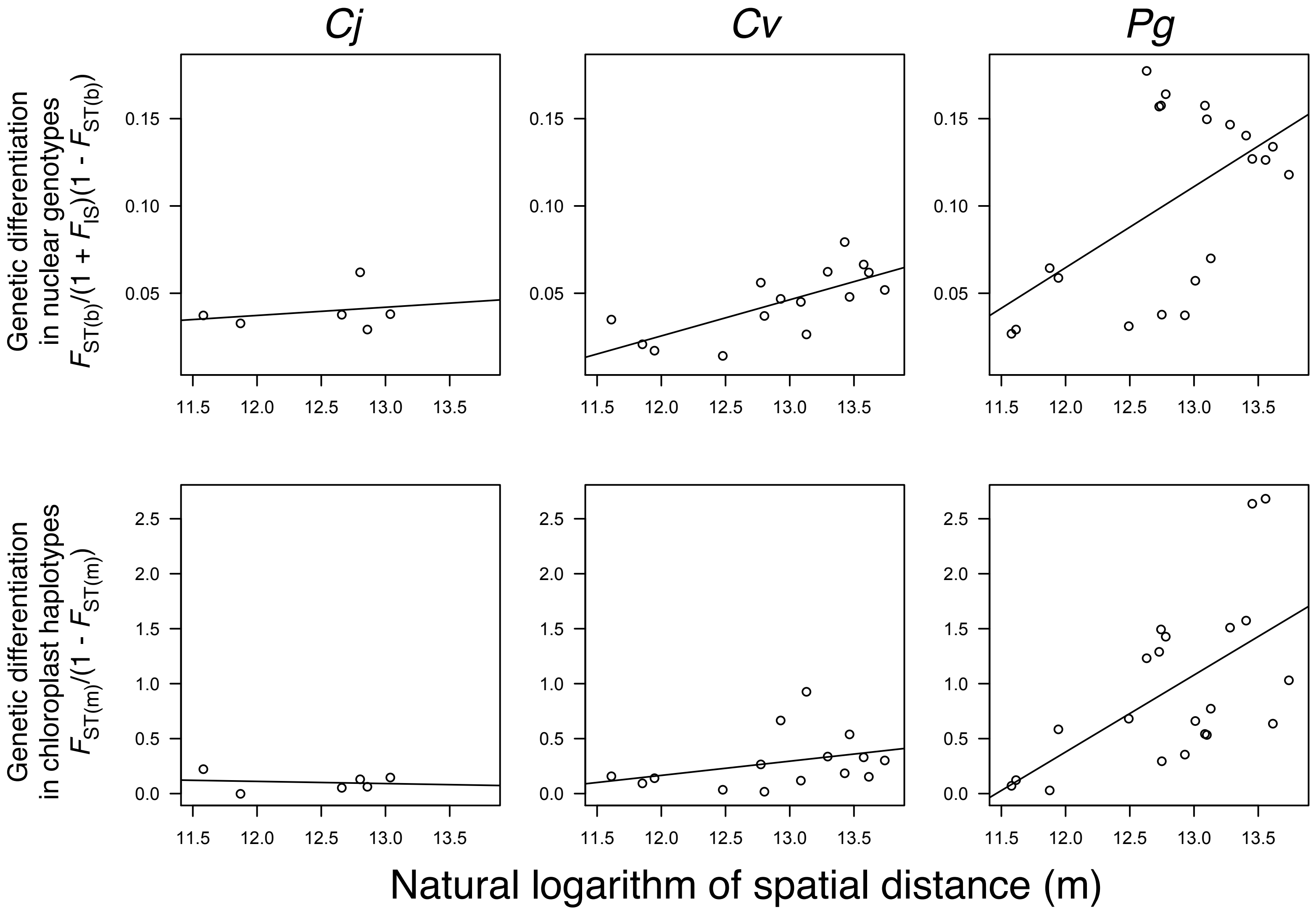


**Fig. S2.** Linear regression lines from natural logarithm of spatial distance to genetic differentiation between populations in nuclear genotypes and chloroplast haplotypes and scatter plots of their relationship in all pairs of study populations of cherry species (*Cj*: *C. jamasakura*, *Cv*: *C. leveilleana*, and *Pg*: *P. grayana*).

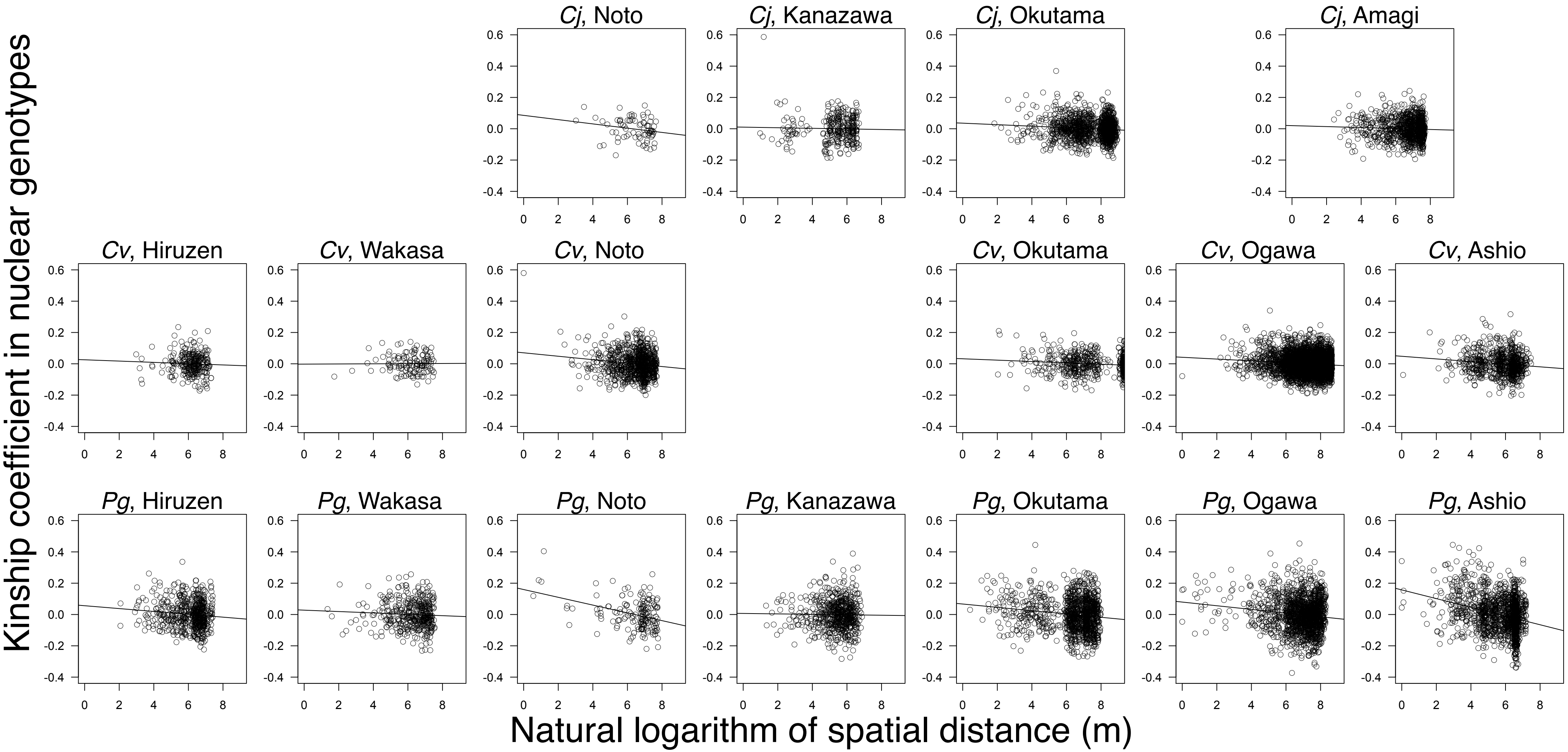


**Fig. S3.** Linear regression lines from natural logarithm of spatial distance to Loiselle's kinship coefficient in nuclear genotypes and scatter plots of their relationship in all pairs of sampled trees in populations of cherry species (*Cj*: *C. jamasakura*, *Cv*: *C. leveilleana*, and *Pg*: *P. grayana*) at study sites.

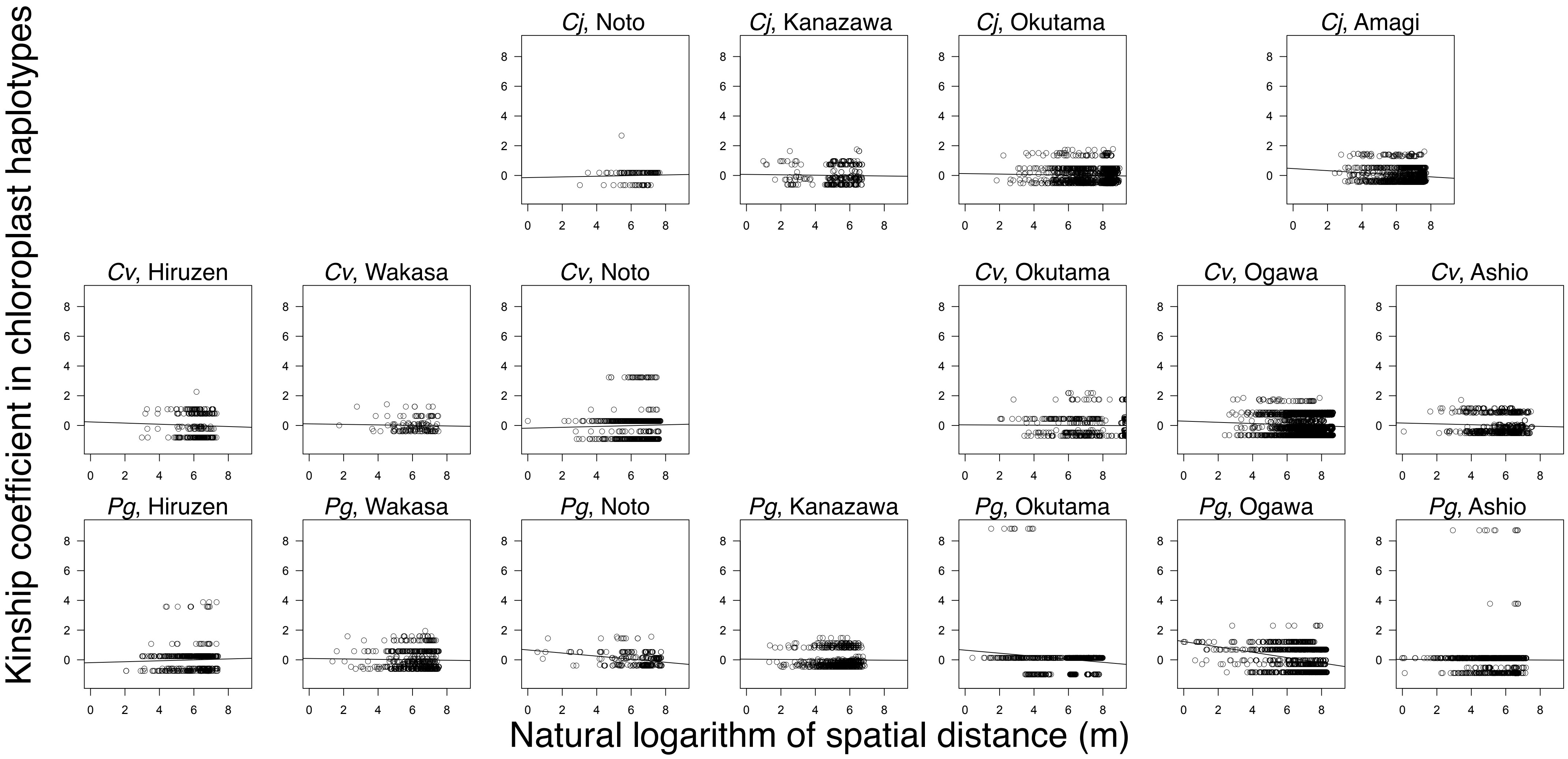


**Fig. S4**. Linear regression lines from natural logarithm of spatial distance to Loiselle's kinship coefficient in chloroplast haplotypes and scatter plots of their relationship in all pairs of sampled trees in populations of cherry species (*Cj*: *C. jamasakura*, *Cv*: *C. leveilleana*, and *Pg*: *P. grayana*) at study sites.
